# Habitat selection in a recovering bobcat (*Lynx rufus*) population

**DOI:** 10.1371/journal.pone.0269258

**Published:** 2022-08-01

**Authors:** Landon R. Jones, Scott A. Johnson, Cassie M. Hudson, Patrick A. Zollner, Robert K. Swihart

**Affiliations:** 1 Department of Forestry and Natural Resources, Purdue University, West Lafayette, Indiana, United States of America; 2 Indiana Department of Natural Resources, Bloomington, Indiana, United States of America; Amity University, INDIA

## Abstract

Understanding habitat selection of top predators is critical to predict their impacts on ecological communities and interactions with humans, particularly in recovering populations. We analyzed habitat selection in a recovering population of bobcats (*Lynx rufus*) in south-central Indiana using a Random Forest model. We predicted that bobcats would select forest habitat and forest edges but avoid agriculture to maximize encounters with prey species. We also predicted that bobcats would avoid developed areas and roads to minimize potential antagonistic interactions with humans. Results partially supported our predictions and were consistent with bobcats in the early stages of population expansion. Bobcats exhibited elevated use near forest edges, thresholds of avoidance near agriculture, and thresholds of selection for low and intermediate habitat heterogeneity. Bobcats exhibited peak probability of use 1–3 km from major roads, >800 m from minor roads, and <1km from developed areas, suggesting tradeoffs in reward for high-quality hunting areas and mortality risk. Our Random Forest model highlighted complex non-linear patterns and revealed that most shifts in habitat use occurred within 1 km of the edge of each habitat type. These results largely supported previous studies in the Midwest and across North America but also produced refinements of bobcat habitat use in our system, particularly at habitat boundaries. Refined models of habitat selection by carnivores enable improved prediction of the most suitable habitat for recovering populations and provides useful information for conservation.

## Introduction

Habitat selection is an emergent property that reflects decisions made by individuals interacting with their environment [1]. Which habitats a species collectively selects, tolerates, or avoids can have far-reaching consequences for interspecific interactions and ecosystems [2–4]. From an ecological perspective, understanding habitat selection is important for species such as mammalian carnivores that are often apex predators and tend to have a disproportionately greater impact on other species in their respective landscapes [5, 6]. From a conservation perspective, habitat selection by top predators has practical implications because it often engenders conflict with humans [7–10]. In particular, understanding habitat selection for species experiencing population recovery after decline is critical, because individuals within a population may use habitat differently during different phases of expansion [7, 11, 12].

Bobcats (*Lynx rufus*), the most widespread felid in North America [13], experienced severe declines and local extirpations in the midwestern U.S. by the mid-1900s [14–16]. Main causes of these declines were overharvest, conversion of forested habitats to cropland, and human persecution [17]. By the mid-1990s, most midwestern states experienced increasing bobcat populations after harvest was halted and the species began to naturally recover [18]. However, from 1970 to 1995 only 7 records were confirmed in Indiana [19]. In the mid-1990s, a remnant population began to expand in forested counties in the south-central portion of the state [17, 19].

To determine habitat selection during recovery of this population, we monitored bobcats from 1998 to 2006 using radio telemetry methods. Based on prior research in the midwestern U.S. in partially recovered populations in Illinois, Iowa, and Michigan [20–22], and recovering populations in Ohio [23, 24] we predicted that bobcats in Indiana would select forest habitat and forest edges but avoid agriculture to maximize encounters with prey species. We also predicted that bobcats would avoid developed areas and roads to minimize antagonistic interactions with humans [20, 22, 25, 26]. We modeled habitat selection by bobcats in our population using Random Forest, which can capture non-linear patterns in habitat selection compared to traditional models [27] and thus aid understanding of bobcat use of habitat boundaries.

## Materials and methods

### Study area

**Fig 1:**
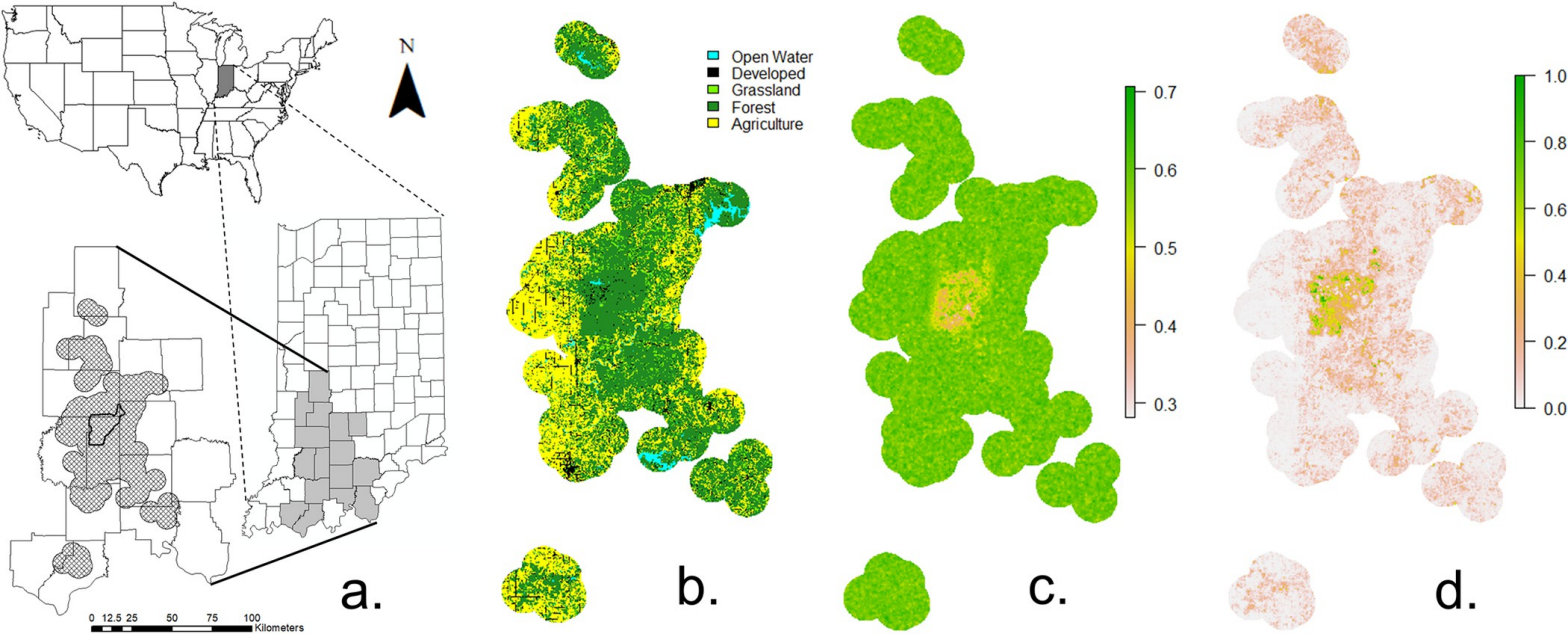
Map of 5,654-km^2^ study area for bobcats (*Lynx rufus*) in south-central Indiana, U.S.A from 1998–2006 (a). The crosshatched area represents the study site within 16 counties in Indiana. The area outlined in black within the study area represents Naval Support Activity Crane. Map of major habitat types (b), habitat heterogeneity values (c), and spatial predictions for the probability of habitat selection at the study area scale (d).

Our study was conducted in the Crawford Upland and Escarpment Sections of the Shawnee Hills Natural Region [28] in south-central Indiana, USA. This rural 5654-km^2^ study area contained portions of 16 counties (Fig 1A). The study area was largely unglaciated, with undulating terrain characterized by low wooded hills, entrenched valleys, and narrow ridge tops. Mature second-growth forests, primarily oak–hickory stands (e.g., *Quercus alba*, *Q*. *rubra*, *Q*. *velutina*, *Carya glabra*, *C*. *ovata*) and mixed upland hardwoods (e.g., *Liriodendron tulipifera*, *Fagus grandifolia*, *Acer saccharum*, *Fraxinus americana*), comprised 54% of the study area [National Land Cover database 2001, 29]. Agricultural crop fields (mainly corn and soybeans) and grasslands (hay fields, pastures) comprised, respectively, 35% and 4% of the study area. The remaining area contained open water (2%) and developed areas (5%), including roads. Bobcat captures were centered on the Naval Support Activity Crane (NSA Crane), a 252-km^2^ military support installation with contiguous blocks of forested habitat.

### Data collection

We trapped and tracked bobcats via radio telemetry from December 1998 to April 2006. Bobcats were trapped during the winters of 1998–2005. After capture, we determined sex and fitted bobcats with a 135 g very high frequency (VHF) radio telemetry collar with a mortality switch (Telonics, Inc., Mesa, AZ) and lined with compressible foam for expansion. Bobcats were anesthetized with an intramuscular injection of a 5:1 mixture of ketamine HCl (KetasetH; Fort Dodge Animal Health, Fort Dodge, IA) and xylazine (Taylor Pharmaceuticals, Decatur, IL) at a dosage of 10 mg/kg of estimated body weight. Further capture methods are described in [17]. Data collection was conducted by personnel from the Indiana Department of Natural Resources. Capture and handling of bobcats followed all ethical guidelines for mammals [30] and all necessary legal permissions for conducting the study were acquired.

We typically relocated collared bobcats 3 days per week using conventional aerial and ground-based telemetry techniques [31]. For ground tracking, we used a 2-element handheld antenna at geo-referenced points to record sequential azimuths within a 15-minute period. We estimated locations using the maximum likelihood estimator in software program LOAS (Location of a Signal; Ecological Software Solutions, Sacramento, CA). Mean error polygon for locations generated by LOAS (*n* = 5761) was 2.7 ha (*SD* = 3.6). We obtained aerial locations using homing techniques from a helicopter equipped with 2 skid-mounted antennae. Error of aerial locations, expressed as the linear distance between estimated and actual coordinates of test collars, averaged 94 m (2.8 ha). We converted locations to Universal Transverse Mercator coordinates (Zone 16, North American Datum 1927) and imported them into a Geographic Information System (GIS) for analyses.

We assessed habitat selection by bobcats at two scales. We used ArcMap 10.5.1 (ESRI, Redlands, CA, U.S.A.) to identify the scale of our study area [2^nd^ order selection, 32] and its habitat characteristics. The spatial extent of our study area was defined by mapping all location estimates for known bobcat points, and buffering each point by the radius (6555 m) of the mean size of all bobcat home ranges [135.0 km^2^, 33], and dissolving lines within overlapping areas [Fig 1A, 34]. To quantify habitat selection within home ranges [3^rd^ order selection, 32], we calculated 95% kernel density estimates (KDE) in the ‘ks’ package in R using the direct plug-in bandwidth selector [35] and converted them to shapefiles. This method performs better when hard boundaries occur in the landscape, such as agricultural fields and hedgerows in our landscape mosaic [36].

To determine habitat selection by bobcats at both scales, we applied a use-availability design to create a resource-selection function [1, 37]. We compared known locations for bobcats to available points randomly selected across our study area and within 95% KDE home ranges. Inadequate sampling of available points compared to known points can provide misleading results for studies of habitat use [38]. Thus, we examined ratios of 1 known to 1, 2, 5, 10, and 20 available bobcat points. Ultimately, we used a 1:10 ratio because that was the minimum ratio at which the mean and standard deviation (*SD*) stabilized across our range of available points for habitat variables (S1 and S2 Figs). We randomly generated all available points at both the study area and within home range scales in ArcMap 10.5.1.

We selected variables to include in the RSFs based on findings from other bobcat studies [13, 20, 22, 25] and available habitat types in southern Indiana. We used nine variables at both scales, including habitat heterogeneity (defined below), gender of bobcats, proximity to five habitat types (forest, agriculture, grassland, developed areas, open water) and proximity to major roads and minor roads (S1 Table). Developed areas comprised human population (or activity) centers that lacked natural habitats. We modified a raster file (30 x 30 m resolution) from the 2001 National Land Cover Database [32] to create our 5 habitat types (Fig 1B) and added road shapefiles for the state of Indiana in ArcMap 10.5.1 (ESRI, Redlands, CA). Forest habitat included deciduous, evergreen, mixed forests, and forested wetlands. Agriculture included hay and cultivated crops, predominantly corn and soybeans. Grassland included herbaceous grasslands, emergent herbaceous wetlands, and shrub-scrub habitats. We considered U.S. highways and state routes as major roads due to higher traffic volumes and speed limits compared to rural county roads, which we considered as minor roads. Major road types were associated with higher mortality risks for bobcats overall compared to minor roads in California and Ohio [26, 39].

Habitat edges, particularly forest edges in the Midwestern U.S., provide bobcats access to additional prey species [13, 20, 22, 25]. Thus, we used proximity covariates to directly quantify ranges of bobcat selection or avoidance at distances to boundaries between each specific habitat type in our RSF. Classifying covariates by this distance-based measure is better suited than habitat categories to identifying edges and also mitigates misclassification of habitats due to triangulation error [40]. Because >57% of used and available locations were in forest, we treated these locations as negative distances to the nearest non-forest edge for this variable to avoid ambiguous interpretation and to better test for the effects of proximity to forest edge in both analyses [41].

Bobcats often use a mix of habitat types for hunting different prey species [13, 20, 22, 25]. Thus, we included in RSFs an index of habitat heterogeneity to account for variation in the composition of habitat types (Fig 1C). For each known and available point, at both study area and home-range scales, we calculated heterogeneity as an index of the proportion of five habitat types (forest, agriculture, open water, grassland, developed areas) within a moving window consisting of a circular buffer equaling the mean size of all bobcat home ranges [135.0 km^2^, radius of 6555 m, 33]. We used the following equation of habitat evenness (*HE_k_*) from [42], based on the Shannon-Weaver index of species diversity [43]:

$${HE}_{k}=-\sum_{i=1}^{n}\frac{\left(P_{i}*ln\;P_{i}\right)}{ln\;n}$$

where *n* equals the number of habitat types in *k* at either the study area or within home ranges scale, and *P_i_* is the proportion of area of habitat *i*. Values for habitat evenness range from 0–1, with 0 representing an area completely covered by a single habitat type, and 1 representing the same area if it were evenly shared by all 5 habitat types.

### Statistical analysis

To assess habitat selection by bobcats in our recovering population, we used a machine-learning approach and fit a Random Forest model [44] using the ‘party’ package in R [45]. We chose a Random Forest modeling framework because we anticipated potential non-linear trends in habitat use by bobcats near habitat boundaries [13]. In addition, our Random Forest models yielded more nuanced and biologically interesting results than generalized linear mixed models (not presented), consistent with results of a comparison of the two modeling approaches for resource selection by mule deer (*Odocoileus hemionus*) in Nevada [27].

We determined the best settings for our Random Forest models based on the lowest values for out-of-bag (OOB) error among a wide range of parameters [27, 46]. For our model at the study area scale, the lowest OOB error occurred with 200 inference trees, a data fraction of 0.623 [default of 0.623, within recommendations of 46], and using 6 of 9 variables selected randomly as the splitting criterion (S2 Table). Within home ranges, the lowest OOB error occurred with the same settings, except that 2 of 9 randomly selected variables were used as the splitting criterion (S3 Table). We created partial dependence plots to visualize the effects of predictors on habitat selection. Finally, we created a spatial map of the study area by applying the Random Forest model to raster maps of each variable except sex to predict relative values for habitat selection (0–1) for each pixel [27].

Interactions were included in our models because Random Forest evaluates univariate and bivariate effects simultaneously and also accommodates multicollinearity of predictor variables [44, 47]. The relative importance of each predictor variable (importance value) includes its contribution to interactions [44, 47] and is computed from random permutation of values of the focal predictor variable for the OOB sample [48]. We explicitly computed interactions among our predictor variables to determine how they may interact at habitat edges. Specifically, we separately calculated and ranked the strength of two-way interactions using root mean squared error (RMSE), according to procedures from [46] and [27]. For each potential two-way interaction, we divided data for each variable into 10 bins, creating a total of 100 bins. Holding other variables constant at mean values, we used the Random Forest model to predict resource selection propensity for each bin. We treated these 100 predictions as the response in a linear model with no interaction terms. We calculated the RMSE from this linear model and used it as an index to rank the focal interaction’s relative importance compared to other interactions, which we calculated in the same manner. The maximum drop in RMSE among ranked interactions was used as a cut-off to select interactions that merited consideration.

*Cross-validation*.–We conducted cross-validation to compare the predictive power of the Random Forest for bobcat resource selection, following [27]. Specifically, we reran the analysis after omitting each individual bobcat from the data set one at a time, and then calculated area under the curve (AUC) using a Receiver Operating Characteristic (ROC) analysis in the ‘ROCR’ package in R [49], to measure predictive skill. Thus, we conducted a total of 54 analyses for both scales of our bobcat study (27 bobcats x 2 scales). All statistical analyses were performed and figures were created in program R 3.5.1 [50].

## Results

We monitored 27 bobcats (14 males, 13 females) and collected 6,958 locations during our study. Used points per individual averaged (± *SD*) 257.7 ± 36.0. At the study-area scale, used points were, on average, farther from agriculture and closer to forest and grassland compared to available points (S1 Table). At the scale of home ranges, used points were closer on average to forest and agriculture (S1 Table).

### Model performance

For the study-area scale, the mean AUC value (± *SE*) across individuals for Random Forest was high (0.86 ± 0.02). However, predictive performance was poor at the scale of the home range, with a mean AUC of 0.59 ± 0.01. Thus, our results indicated that model fit was too poor to justify further exploration of habitat selection by bobcats at the home-range scale.

### Study-area scale

**Fig 2:**
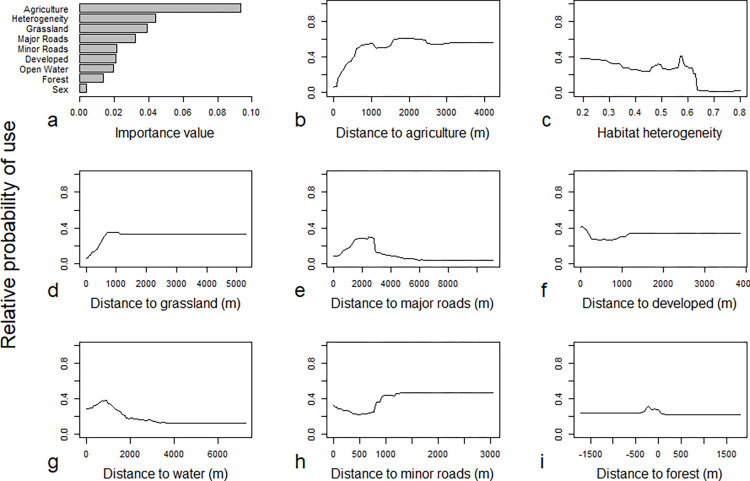
Importance variables (a) and partial dependence plots (b–i) for main effects, ranked by importance values, characterizing habitat selection at the scale of the study area in Random Forest analyses for bobcats (*Lynx rufus*) in south-central Indiana, U.S.A. from 1998–2006.

The top four variables, ranked in order of importance value, were agriculture, heterogeneity, grassland, and major roads (Fig 2A). The importance value for agriculture was more than twice that of heterogeneity, the predictor with the next highest importance value (Fig 2A). The remaining five variables had importance values approximately half or less than values for the four most important predictors (Fig 2A). The importance value for forest was second to last among predictors (Fig 2A). Two interactions (agriculture and heterogeneity, agriculture and developed) were also important, according to RMSE values (S4 Table).

**Fig 3:**
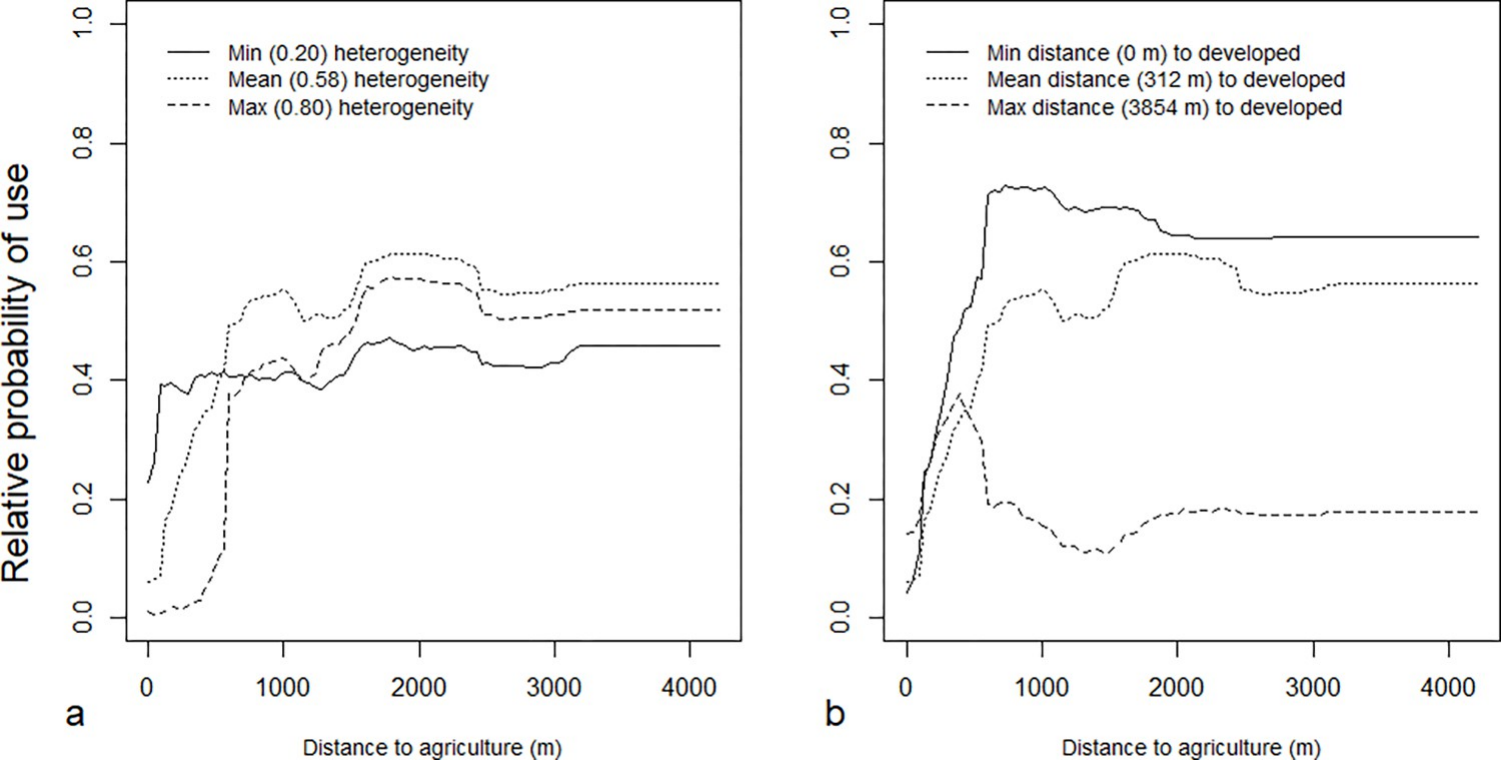
Partial dependence plots for interactions with agriculture and significant variables characterizing habitat selection at the scale of the study area for Random Forest analyses for bobcats (*Lynx rufus*) in south-central Indiana, U.S.A. from 1998–2006.

Partial dependence plots revealed complex patterns, such as thresholds and plateaus in use, often <1 km from habitat boundaries (Figs 2 and 3). Bobcat habitat use typically was low near agriculture, increased rapidly as distance to agriculture increased to ~1 km (Fig 3), and was more common in areas with average or above average habitat heterogeneity (Fig 3A). Probability of use increased and then plateaued at approximately 800 m from grassland (Fig 2D) and peaked 1–3 km from major roads (Fig 2E). Contrary to expectations, probability of use was slightly more common near developed areas than far from them (Fig 2F). Peaks in probability of use occurred at approximately 1 km from open water (Fig 2G) and >800 m from minor roads (Fig 2H). Use of forest was characterized by a range of elevated values from 300 m inside forest to 50 m outside of it, with a peak at approximately 225 m within forest (Fig 2I).

Two interactions further characterized the effects of distance to agriculture, the most important predictor of habitat use by bobcats. The interaction of agricultural proximity and habitat heterogeneity showed that propensity of use for low habitat heterogeneity, relative to high and mean heterogeneity, switched rapidly from greatest to least over a short range (approximately 500–700 m) of distances to agriculture (Fig 3A). The interaction of proximity to agriculture and development predicted uniformly low use near agriculture, followed by peak use closer to agricultural edges (~500 m) and stabilization at lower levels (~0.2) far from development compared to predictions closer to development (Fig 3B).

### Spatial projections of resource selection

Although forest and agriculture were the most prevalent habitat types in our landscape (Fig 1C), only distance to agriculture was important in the model. In particular, habitat heterogeneity was more important than forest proximity. Low and intermediate values of habitat heterogeneity were concentrated in large blocks of forest sharing fewer edges with other habitat types (Fig 1C). At the scale of the study area, spatial predictions of increased probability of habitat use by bobcats were generally associated with forested areas and avoided agricultural areas (Fig 1D). Spatial predictions showed that overall, larger blocks of forest contained high probabilities of use (Fig 1D). However, the highest probabilities were in large, forested blocks on NSA Crane and adjacent areas (Fig 1D), more closely mirroring areas of low habitat heterogeneity (Fig 1C).

## Discussion

Habitat selection by a recovering population of bobcats in south-central Indiana largely supported previous studies in the Midwest and across North America [13, 20–22] but also produced refinements in habitat use, particularly at habitat boundaries. Bobcats selected forest and forest edges and avoided agriculture, supporting our first prediction and consistent with previous studies [20–22]. However, only partial support emerged for our second prediction that bobcats would avoid developed areas and roads. Similar to other studies, bobcats in our population avoided minor roads [20–22]. In contrast to populations in other areas, our bobcats tended to select locations nearer developed land cover types and at intermediate distances to major roads [25, 39, 51]. Our Random Forest model highlighted complex non-linear patterns and revealed that most shifts in habitat use occurred within 1 km of the edge of each habitat type.

Bobcat selection of forest edges is well documented [13, 20–22]. Bobcats use these features to ambush prey, especially rabbits (*Sylvilagus*), small rodents, and avian species [13]. Our Random Forest results predicted a range of peak use from 300 m within to 50 m outside of forest, providing additional quantitative information delineating the spatial range over which habitat selection occurred along forest edges in our system.

Avoidance of agriculture and selection for intermediate and low heterogeneity (calculated within moving circular windows the size of a bobcat home range) were the primary drivers of habitat selection in our landscape. Thresholds and plateaus predicted by Random Forest also improved our understanding of use by bobcats for these landscape predictors. At >500 m from agriculture, Random Forest predicted plateaus of higher use associated with areas of low and intermediate habitat heterogeneity. Maps of habitat types and heterogeneity showed that low-heterogeneity regions were predominantly concentrated in large forest blocks on or near NSA Crane. Accordingly, spatial predictions showed the highest probabilities of use for bobcats in this area of forested habitat and lower values in forested tracts outside of low-heterogeneity areas. These results are supported by a previous study in adjacent Illinois; use of smaller forest fragments surrounded by agriculture or other habitat types is less commonly documented for bobcats and was associated with low-density populations [52].

Indiana’s bobcat population during our study period was recovering and at low density [19]. Selection of low heterogeneity areas by bobcats in our population likely reflected their selection of the highest quality habitat, which presumably was more widely available to the individuals at our study site. Our results differed from a statewide model of bobcat habitat suitability in Indiana that was developed using similar predictors and presence-only data from 2010–2020 [53]. A critical distinction is that this statewide model was based on data from a time when population trends indicated bobcats were increasing rapidly and appeared to eventually reach stable and high numbers across the state [19]. In the data used for this statewide model, forest represented only 22.9% of Indiana’s landscape. The most important predictors for habitat suitability in this statewide model were intermediate values for proportion of natural habitats (forest, wetlands, grasslands) < 4 km from bobcat locations, and close proximity to forest [53]. Beyond differences in data type and analyses, another key distinction between the statewide model and the results of this study in south-central Indiana is that this study was conducted when Indiana’s bobcat population size was low. Habitat selection studies on other carnivores suggest that recolonizing populations occupy habitat of highest quality first, followed by use of lower quality habitats after population expansion. Such trends have been documented for Eurasian lynx (*Lynx lynx*) in Sweden [12], wolves (*Canis lupus*) in the north-central U.S. [7], and Eurasian otters (*Lutra lutra*) in the Iberian Peninsula [11]. Our results are consistent with these patterns.

Understanding bobcat probability of use for roads in our system was improved by the Random Forest model compared to previous studies [26, 39, 54, 55]. For example, Random Forest identified a range of peak use 1–3 km from major roads and trough of low habitat use 0–800 m from minor roads. Our results likely represent ranges of bobcat tolerances to these road types due to mortality risks [39, 51]. The peak in use at intermediate distances to major roads may indicate a tradeoff between decreased foraging opportunities >3 km from major roads and avoidance of roads within 0.5 km due to mortality risks [23, 39]. Other carnivores have demonstrated the same tradeoff in different contexts, selecting increased reward in high-quality hunting or foraging areas and avoiding mortality risks near roads associated with humans [56–58]. For example, Eurasian lynx established home ranges within areas of high prey and road densities but avoided roads within home ranges [56]. Wolves and grizzly bears (*Ursus arctos*) seasonally select areas near forest roads to improve access to prey or fruiting resources but avoid the same areas due to human presence in other seasons [57, 58].

Surprisingly, bobcats in our study tended to select locations nearer developed areas. Previous studies indicated that bobcats typically avoid developed areas and humans [13, 51, 52]. Our results likely were driven by land management on NSA Crane, where most bobcats were trapped and Random Forest predicted the highest-quality habitat. Developed areas at Crane included many buildings near forested habitat such as storage facilities that were infrequently used by humans compared to other areas of the base. The edges for many of these developed areas were consistently mowed and maintained by base personnel, creating open areas abutting forest. Such habitat edges are ideal habitat for bobcats due to high availability of prey such as rabbits [13]. Structural resources can also affect predation near habitat edges [59]. In particular, bobcats use forest structure to provide concealment when ambushing prey [13], which could enhance the value of edges near developed sites on Crane. Anecdotally, we regularly observed bobcats in these areas at Crane. A few studies have shown higher-than-expected bobcat densities in urban or suburban areas [39, 60], suggesting some level of tolerance of humans where habitat is suitable. Still other felid species including Eurasian lynx, cougars, and African lions select areas nearer human development at night, when risk of human detection is low compared to daytime [61–63]. At the scale of home ranges, predictive skill was poor for our model, possibly because one or more important predictor variables were missing from our models. Alternatively, the precision of our bobcat location estimates (2.7 ha) and resulting habitat classification errors could have been too coarse to allow meaningful home-range scale inference. The distance-based approach we used to estimate habitat predictors tends to mitigate misclassification of habitats due to triangulation error [40], but it may have been insufficient at the home-range scale for our study. In particular, the 30 x 30 m landcover data may not have provided sufficient resolution to observe selection of habitat features like gaps within forest [64]. Consequently, we could not test if our bobcats selected forest openings within their home ranges. Forest openings provided the highest bobcat densities and smallest home ranges in Alabama [65]. Similarly, bobcats in the Appalachian mountains selected canopy openings and avoided the forest interior within home ranges [66].

Understanding complex patterns of habitat selection by carnivores enables improved prediction of the most suitable habitat for recovering populations [12, 67] and provides useful information for carnivore conservation [6, 68]. For bobcats in south-central Indiana, Random Forest models revealed specific thresholds and ranges of habitat use at forest and anthropogenic boundaries that were consistent with previous studies and with an expanding population. Our results imply that bobcats in our study area would benefit most from maintenance of forested areas furthest from other habitat types. Additionally, our results indicate that bobcats view anthropogenic boundaries such as roads, development, and agriculture in terms of context-dependent tradeoffs and not as simple selection or avoidance of these features [39, 51]. Such complex behavioral tradeoffs likely apply more broadly to other carnivore species, especially those inhabiting human-dominated landscapes [57, 61, 62].

## Supporting information

S1 Fig Mean values for distance to habitat types per individual bobcat (*Lynx rufus*) for number of pseudo-absence points (available) per radio telemetry point (used) for 27 bobcats at the scale of the study area in south-central Indiana, U.S.A. from 1998–2006. (DOCX)

S2 Fig Mean values for distance to habitat types per individual bobcat (*Lynx rufus*) for number of pseudo-absence points (available) per radio telemetry point (used) for 27 bobcats at the home range scale in south-central Indiana, U.S.A. from 1998–2006. (DOCX)

S1 Table Mean, standard deviation (*SD*), minimum (Min), and maximum (Max) values for nine variables in habitat selection analyses for used (animal locations) and available points at two scales (study area, home range) for bobcats (*Lynx rufus*) in south-central Indiana, U.S.A. from 1998–2006. (DOCX)

S2 Table Out of bag error (OOB) across data ranges for three parameters, number of trees (ntree), number of variables used (mtry), and data fraction used (fraction), in a Random Forest analysis at the scale of the study area for bobcats (*Lynx rufus*) in south-central Indiana, U.S.A. from 1998–2006. Final values for each variable were selected based on the lowest value with the smallest OOB value (*) or the default value if values were similar across the range and no trend was apparent (fraction, 0.623).
(DOCX)

S3 Table Out of bag error (OOB) across data ranges for three parameters, number of trees (ntree), number of variables used (mtry), and data fraction used (fraction), in a Random Forest analysis at the scale of home ranges for bobcats (*Lynx rufus*) in south-central Indiana, U.S.A. from 1998–2006. Final values for each variable were selected based on the lowest value with the smallest OOB value (*) or the default value if values were similar across the range and no trend was apparent (fraction, 0.623).
(DOCX)

S4 Table Root mean squared error (RMSE) values, ranked from highest to lowest, for all potential interactions in a Random Forest model for nine variables in a habitat selection analysis for bobcats (*Lynx rufus*) in south-central Indiana, U.S.A. from 1998–2006. (DOCX)
