# Supplementary material for: Habitat selection in a recovering bobcat (Lynx rufus) population
Source: PLoS One. 2022 Aug 1;17(8):e0269258. doi: 10.1371/journal.pone.0269258 (PMC9342758; doi:10.1371/journal.pone.0269258)

**Figure S2.** Mean values for distance to habitat types per individual bobcat (*Lynx rufus*) for number of pseudo-absence points (available) per radio telemetry point (used) for 27 bobcats at the home range scale in south-central Indiana, U.S.A. from 1998-2006.


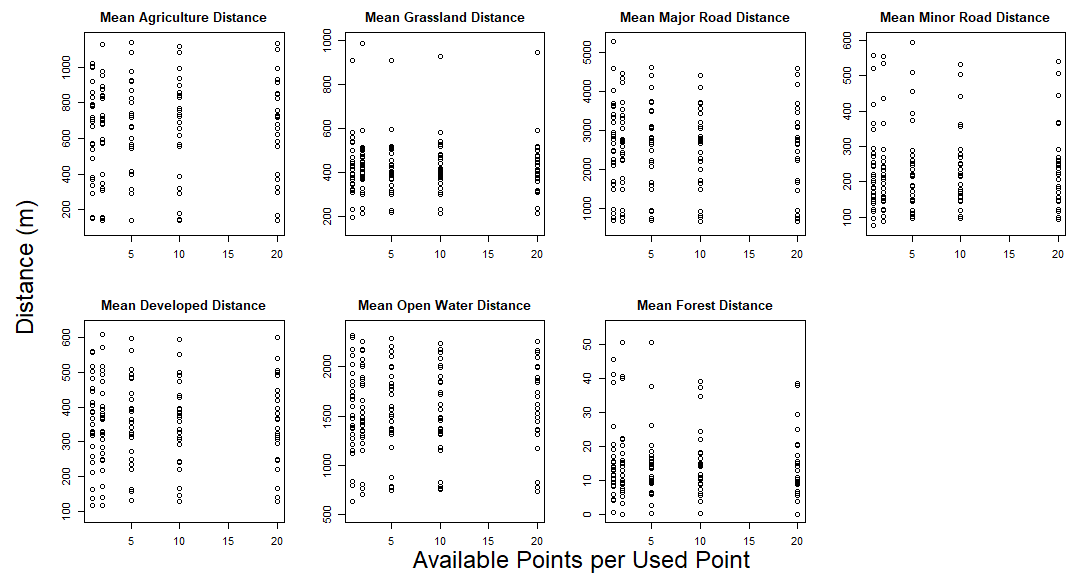

Supplement: S2 Fig — (DOCX) [file pone.0269258.s002.docx]
