# Supplementary material for: Habitat selection in a recovering bobcat (Lynx rufus) population
Source: PLoS One. 2022 Aug 1;17(8):e0269258. doi: 10.1371/journal.pone.0269258 (PMC9342758; doi:10.1371/journal.pone.0269258)
Supplement: S1 Table — (DOCX) [file pone.0269258.s003.docx]

**Table S1.** Mean, standard deviation (*SD*), minimum (Min), and maximum (Max) values for nine variables in habitat selection analyses for used (animal locations) and available points at two scales (study area, home range) for bobcats (*Lynx rufus*) in south-central Indiana, U.S.A. from 1998-2006.

|  |  |  |  |  | Study area scale | | | | Home range scale | | | |
| --- | --- | --- | --- | --- | --- | --- | --- | --- | --- | --- | --- | --- |
|  | Used points | | | | Available points | | | | Available points | | | |
| Variable | Mean | *SD* | Min | Max | Mean | *SD* | Min | Max | Mean | *SD* | Min | Max |
| agriculture | 730.0 | 718.3 | 0.0 | 4214.1 | 260.5 | 447.9 | 0.0 | 4216.4 | 770.7 | 775.7 | 0.0 | 4266.8 |
| developed | 358.3 | 281.5 | 0.0 | 1643.4 | 307.4 | 270.5 | 0.0 | 3853.7 | 342.2 | 280.7 | 0.0 | 1718.1 |
| forest | -108.8 | 126.2 | -823.8 | 324.5 | 6.6 | 198.3 | -1717.4 | 1798.2 | -90.6 | 132.9 | -829.8 | 674.2 |
| grassland | 412.2 | 311.4 | 0.0 | 1972.0 | 528.9 | 635.4 | 0.0 | 5294.3 | 411.2 | 321.7 | 0.0 | 3378.4 |
| open water | 1535.7 | 912.9 | 0.0 | 6085.6 | 1415.6 | 1170.3 | 0.0 | 7240.1 | 1533.9 | 929.7 | 0.0 | 6592.2 |
| major roads | 2533.5 | 1669.9 | 0.0 | 8578.3 | 2406.1 | 1924.3 | 0.0 | 11128.4 | 2649.7 | 1777.7 | 0.0 | 8828.1 |
| minor roads | 231.0 | 226.4 | 0.0 | 1530.0 | 257.6 | 226.1 | 0.0 | 3053.7 | 214.3 | 213.7 | 0.0 | 1560.3 |
| heterogeneity | 0.5 | 0.1 | 0.2 | 0.8 | 0.6 | 0.1 | 0.4 | 0.8 | 0.5 | 0.1 | 0.2 | 0.8 |
| sex | 0.4 | 0.5 | 0.0 | 1.0 | 0.4 | 0.5 | 0.0 | 1.0 | 0.4 | 0.5 | 0.0 | 1.0 |
