# Supplementary material for: Habitat selection in a recovering bobcat (Lynx rufus) population
Source: PLoS One. 2022 Aug 1;17(8):e0269258. doi: 10.1371/journal.pone.0269258 (PMC9342758; doi:10.1371/journal.pone.0269258)
Supplement: S2 Table — Final values for each variable were selected based on the lowest value with the smallest OOB value (*) or the default value if values were similar across the range and no trend was apparent (fraction, 0.623). (DOCX) [file pone.0269258.s004.docx]

**Table S2.** Out-of-bag error (OOB) across data ranges for three parameters, number of trees (ntree), number of variables used (mtry), and data fraction used (fraction), in a Random Forest analysis at the scale of the study area for bobcats (*Lynx rufus*) in south-central Indiana, U.S.A. from 1998-2006. Final values for each variable were selected based on the lowest value with the smallest OOB value (*) or the default value if values were similar across the range and no trend was apparent (fraction, 0.623).

| ntree | OOB | mtry | OOB | fraction | OOB |
| --- | --- | --- | --- | --- | --- |
| 10 | 5.10 | 2 | 3.96 | 0.1 | 3.53 |
| 50 | 3.78 | 3 | 3.69 | 0.2 | 3.54 |
| 100 | 3.66 | 4 | 3.58 | 0.3 | 3.56 |
| 200 | 3.64* | 5 | 3.57 | 0.4 | 3.55 |
| 300 | 3.68 | 6* | 3.54 | 0.5 | 3.54 |
| 400 | 3.67 | 7 | 3.54 | 0.6 | 3.54 |
| 500 | 3.68 | 8 | 3.55 | 0.7 | 3.54 |
| 750 | 3.69 | 9 | 3.57 | 0.8 | 3.53 |
| 1000 | 3.66 |  |  | 0.9 | 3.53 |
