# Supplementary material for: Habitat selection in a recovering bobcat (Lynx rufus) population
Source: PLoS One. 2022 Aug 1;17(8):e0269258. doi: 10.1371/journal.pone.0269258 (PMC9342758; doi:10.1371/journal.pone.0269258)
Supplement: S3 Table — Final values for each variable were selected based on the lowest value with the smallest OOB value (*) or the default value if values were similar across the range and no trend was apparent (fraction, 0.623). (DOCX) [file pone.0269258.s005.docx]

**Table S3.** Out-of-bag error (OOB) across data ranges for three parameters, number of trees (ntree), number of variables used (mtry), and data fraction used (fraction), in a Random Forest analysis at the scale of home ranges for bobcats (*Lynx rufus*) in south-central Indiana, U.S.A. from 1998-2006. Final values for each variable were selected based on the lowest value with the smallest OOB value (*) or the default value if values were similar across the range and no trend was apparent (fraction, 0.623).

| ntree | OOB | mtry | OOB | fraction | OOB |
| --- | --- | --- | --- | --- | --- |
| 10 | 11.86 | 2* | 9.57 | 0.1 | 9.58 |
| 50 | 9.95 | 3 | 9.82 | 0.2 | 9.54 |
| 100 | 9.91 | 4 | 9.88 | 0.3 | 9.58 |
| 200 | 9.81* | 5 | 9.97 | 0.4 | 9.59 |
| 300 | 9.83 | 6 | 9.98 | 0.5 | 9.57 |
| 400 | 9.81 | 7 | 9.97 | 0.6 | 9.56 |
| 500 | 9.81 | 8 | 10.03 | 0.7 | 9.57 |
| 750 | 9.82 | 9 | 9.99 | 0.8 | 9.55 |
| 1000 | 9.8 |  |  | 0.9 | 9.59 |
