# Supplementary material for: Habitat selection in a recovering bobcat (Lynx rufus) population
Source: PLoS One. 2022 Aug 1;17(8):e0269258. doi: 10.1371/journal.pone.0269258 (PMC9342758; doi:10.1371/journal.pone.0269258)
Supplement: S4 Table — (DOCX) [file pone.0269258.s006.docx]

**Table S4.** Root mean squared error (RMSE) values, ranked from highest to lowest, for all potential interactions in a Random Forest model for nine variables in a habitat selection analysis for bobcats (*Lynx rufus*) in south-central Indiana, U.S.A. from 1998-2006.

| Variable | RMSE |
| --- | --- |
| agriculture x heterogeneity | 0.066 |
| agriculture x developed | 0.064 |
| agriculture x minor roads | 0.055 |
| heterogeneity x open water | 0.053 |
| forest x open water | 0.052 |
| agriculture x grassland | 0.045 |
| major roads x minor roads | 0.044 |
| heterogeneity x major roads | 0.041 |
| heterogeneity x forest | 0.040 |
| heterogeneity x grassland | 0.036 |
| grassland x major roads | 0.033 |
| major roads x open water | 0.029 |
| forest x developed | 0.029 |
| forest x minor roads | 0.028 |
| agriculture x major roads | 0.026 |
| heterogeneity x developed | 0.026 |
| heterogeneity x minor roads | 0.026 |
| major roads x developed | 0.024 |
| minor roads x open water | 0.023 |
| agriculture x open water | 0.022 |
| grassland x open water | 0.022 |
| forest x major roads | 0.022 |
| grassland x developed | 0.018 |
| heterogeneity x sex | 0.016 |
| developed x open water | 0.014 |
| agriculture x sex | 0.014 |
| developed x minor roads | 0.012 |
| forest x grassland | 0.011 |
| minor roads x sex | 0.011 |
| grassland x minor roads | 0.010 |
| agriculture x forest | 0.009 |
| major roads x sex | 0.008 |
| developed x sex | 0.006 |
| forest x sex | 0.006 |
| grassland x sex | 0.005 |
| open water x sex | 0.003 |
